# Individual characteristics on multicultural team performance: does the role played by leaders and team members matter?

**DOI:** 10.3389/fpsyg.2023.1281422

**Published:** 2023-12-19

**Authors:** Laura Esmeralda Guzmán-Rodríguez, Amaia Arizkuren-Eleta, Tanuja Agarwala, Mar Bornay-Barrachina

**Affiliations:** ^1^Monterrey Institute of Technology and Higher Education, Monterrey, Mexico; ^2^Deusto Business School, University of Deusto, San Sebastian, Spain; ^3^Faculty of Management Studies, University of Delhi, Delhi, India; ^4^Management Department, University of Cádiz, Jerez-Cádiz, Spain

**Keywords:** leader role, multicultural team, team performance, cultural sensitivity, adaptability, cohesion, cultural diversity

## Abstract

The main purpose of this study was to analyze the impact of individual characteristics of cultural sensitivity, adaptability, cohesion, and cultural diversity on the multicultural team performance. Also, we analyzed the moderating effect of being a team member or a team leader on the relationships mentioned above. To test out hipotheses, data were collected from 415 members of multicultural teams specifically, from 304 team members and 111 leaders. The findings provided evidence to support a positive relationship between cultural sensitivity, adaptability, cohesion, and team performance. Cultural diversity did not show a significant impact on team performance. The study also showed that the leaders and members had different perceptions about the factors that influence team performance. For instance, team members consider that cohesion influences team performance, while leaders consider adaptability as the key factor to achieve performance. Main implications from findings are discussed.

## 1 Introduction

With today’s highly heterogeneous workplaces, the growing need for companies to include cultural diversity as a characteristic in their workforce (Gonçalves et al., 2020) have made multicultural teams increasingly being used by companies as one of the key ways of collaboration in order to increase productivity (Choi et al., 2018; Ratasuk and Charoensukmongkol, 2020) and achieve organizational goals (Hoever et al., 2012).

Literature shows a large list of benefits that multicultural teams bring to companies, in terms of creativity (Ali et al., 2019), innovation (Jones et al., 2020), competitiveness (Neukam, 2017), organizational commitment (Stahl and Maznevski, 2021), problem solving (Yasmeen et al., 2020), and improved decision making (Han and Beyerlein, 2016; Neukam, 2017).

Culturally diverse teams (multicultural teams) are considered a key source of organizational success and to achieve organizational performance (Ho et al., 2017). The mostly studies were focused on single variables such a demographic diversity, informational diversity, language, or management, among others, and specifically, demographic diversity has been one of the most popular factor. However, studies showed conflicting results, for instance, Thomas (1999) found a negative relationship between cultural diversity and team performance, while Cox and Blake (1991) and Gibson (1999) found a positive relationship. Earley and Mosakowski (2000) found a curvilinear relationship and Williams and O’Reilly (1998) found no relationship between both variables. Socio-cultural background and identity differences frequently result in disagreements and mistrust in culturally team dynamics (Van Knippenberg and Mell, 2016). Research regarding the diverse factors that influence team functioning and performance (Boone et al., 2019; Salzberg et al., 2019) has never been more necessary (Stahl and Maznevski, 2021). Explicitly, at the top management team level, Boone et al. (2019, p.278) stated that “*TMT nationality diversity received only limited attention in prior research and we still know little about why and when it affects innovation performance of MNCs*.” Therefore, there is still lack of knowledge about how many factors influence the team performance, specifically in multicultural contexts, and the way in which they do it. Therefore, we attempt to contribute to this branch of study by examining characteristics of multicultural team members and leaders, namely cultural sensitivity, adaptability, cohesiveness and cultural diversity as key factors in determining team performance.

Additionally, previous literature has pointed out that sometimes there exists disparity in perceptions of important organizational issues between leaders and the team members they lead (Gibson et al., 2009; Tafvelin et al., 2017). Differences in the views have implications for team performance (Kline, 2001). We also contribute to the limited research on study of differences in perceptions among team members and team leaders regarding what is relevant for team performance (Tafvelin et al., 2017; Tuncdogan et al., 2017). As noted by Ratasuk and Charoensukmongkol (2020), dissimilarity in perceptions may negatively impact team performance.

McLeod et al. (1996) established that a successful team performance takes place when the team achieves the established objectives. In general terms, a high performing team will exhibit positive engagement in taskwork and teamwork behaviors, involving shared integration, synthesis and sharing of information (Salas et al., 2008). In a context of diversity and multiculturality, compared to teams composed of members from one culture, multicultural teams can achieve better and more effective performance due to the set of values, characteristics, capabilities, and emotions of each team member (Salas et al., 2008) achieving high results.

Specifically, for this study, multicultural team performance will be defined as the result achieved by the team that meet the required productivity (Tabassi et al., 2017, 2019), quality, and time standards (Kirkman and Shapiro, 2001; West and Markiewicz, 2004; Lawler and Worley, 2006).

## 2 Cultural sensitivity and multicultural team performance

The importance of cultural sensitivity in interacting with individuals from different cultures (Horverak et al., 2013; Lefringhausen et al., 2020) is a relevant variable in any multicultural team. There are studies stating that individuals who possess cultural intelligence establish open and tolerant attitudes by improving performance levels in a multicultural team (Ang et al., 2006).

Terrell and Rosenbusch (2013) define cultural sensitivity as a set of skills that involves understanding and managing cultural differences and having the willingness and an open and inclusive attitude to analyze members’ relationships from different perspectives. Cheng et al. (2012) and Lee et al. (2021) believe that all team members should receive adequate training in cultural sensitivity to solve problems arising from cultural differences that may affect team performance positively. Handin and Steinwedel (2006) add that the perception and appreciation of cultural differences by the leader and team members allows for meaningful relationships with people from other cultures. Therefore, a high level of cultural sensitivity in both the leader and the members of a team facilitates the effective performance of the team (Van der Zee and van Oudenhoven, 2000).

Terrell and Rosenbusch (2013) and Cumberland et al. (2016) state that global leaders must have skills in relating to people from different cultures, as it is a determining role for team performance. Likewise, Puck et al. (2008) and Aretoulis (2018) argue that cultural sensitivity allows individuals in a team to recognize and identify cultural differences among members, which allows them to improve their work together and achieve the objectives set. Kappagomtula (2017) states that if team members and leaders are aware of the cultural differences of their teammates, they can establish a closer relationship with them, generating greater trust and facilitating creative collaboration.

Therefore, according to the literature review established on cultural sensitivity, it is observed that this variable positively affects the team’s performance. Based on the above, the following hypothesis is proposed:

*H1:* Cultural sensitivity is positively related to multicultural team performance.

## 3 Adaptability and multicultural team performance

According to the literature, the term of “team adaptation” is defined as the result of one or several changes that leads to effective team performance (Beal et al., 2003).

To achieve successful performance, teams must adapt. Such adaptation involves, on the one hand, team-level skills such as cohesion, interaction norms, goal clarity (Kozlowski et al., 1999), and group learning (Ramos-Villagrasa et al., 2019) and, on the other hand, individual skills of team members such as knowledge sharing (Bedwell, 2019; Park and Park, 2019), learning orientation, self-regulation (Ramos-Villagrasa et al., 2019), personal bonds, social interactions with local colleagues, national colleagues and family members (Kang and Shen, 2018; Bayraktar, 2019), and similarity of cultures (Varma et al., 2020).

At the individual level, adaptability is defined as work behavior that helps employees adapt to change by demonstrating excellence in problem-solving, uncertainty management, stress, crisis, new learning, and adaptability related to people, culture, and environment (Pulakos et al., 2000; Park and Park, 2019). This ability to adapt is considered one of the strategic global talent development skills required to do business effectively (Bayraktar, 2019). Meanwhile, Beus et al. (2014) refer to adaptation as “the ability to perform tasks satisfactorily and in a short time after joining the team” (p.490), overcoming difficulties experienced in daily work (Konanahalli et al., 2014). Organizations that foster the ability to adapt, their employees present better levels of individual performance and successful change management and promote organizational learning (Park and Park, 2019).

Taking into account the cultural component of the multivultural teams, the cultural adaptability of teams is a phenomenon that has been studied in the literature on expatriate management (Puck et al., 2008; Chen et al., 2010; Harari et al., 2018). McNulty and Brewster (2017) define cultural adaptation as the degree of comfort or absence of stress experienced by the expatriate when participating in an international team. House et al. (2004) provide another definition of cultural adaptability, considering it as the ability to understand other cultures and behave accordingly in order to achieve established goals and establish a positive relationship with peers and team leaders. The process cultural adaptation occurs in two stages: an exchange of knowledge and management of cultural differences where the ability of individuals to adapt their behavior to a specific cultural context plays an essential role (Javidan et al., 2006).

One of the best known models of cultural adaptation is the model by Black et al. (1991) which states that cultural adaptation is a process composed of three elements: the cultural adaptation of living in the host country, the interaction with people from the host culture and the job responsibilities of the new job. Puck et al. (2008) and Takeuchi et al. (2019) consider expatriate adaptation as a relevant benchmark for measuring a member’s adaptation to a multicultural team.

There are several studies (Lawson et al., 2009; Lu and Tjosvold, 2013; Low et al., 2020) on the adaptation of new members to a team; socialization practices between the new member and team members are vital for establishing positive links that lead to a rapid adaptation in the team and, to a greater extent, in a multicultural team. Beus et al. (2014) argue that bringing a new team member on to the team creates uncertainty for both the member and the team, which can affect performance. Bouncken et al. (2016) argue that the experience in cultural adaptability of both the team and the new member are elements that favor the rapid and positive integration of the team member. Following the above mentioned arguments, team member adaptability can make grow the degree of comfort when working together leader, members coming from different cultures, increasing the problem-solving capability, reducing uncertainty and stress, enhancing new learning, and as a concequence, and improving team performance. Therefore, the following hypothesis is proposed:

*H2:* Adaptability is positively related to multicultural team performance.

## 4 Cohesion and multicultural team performance

Cohesion is a relevant variable that represents a shared perception and experience among team members (Carron et al., 2002; Kozlowski and Chao, 2012). The term *team cohesion* is often defined as the mutual bond of attraction that forms between the members of a group, resulting from working for a common purpose and backed by the intention to remain united (Keyton and Springston, 1990; Casey-Campbell and Martens, 2009). Therefore, cohesion is a relevant variable to measure team performance (Salas et al., 2015) since individuals are critical drivers when working in teams (Judge and LePine, 2007).

Weiss et al. (2017) explain the importance of the study of cohesion for the team performance, describing how the process of selection of individuals should be. Acton et al. (2020) study the impact that team composition has on cohesion, indicating that a bad team composition generates a negative cohesion and therefore, harms the team performance level.

Mach et al. (2010) explain that trust and team cohesion play a mediating role, explaining that trust among teammates mediates the relationship between trust in the leader and team cohesion affecting team performance. Rodríguez-Sánchez et al. (2017) analyze the relationship between cohesion and performance (creative and perceived) in a reciprocal way through the mediating effect of collective commitment. The results show that creative teams tend to develop a strong cohesion that leads to better performance.

Previous studies point out the importance of analyzing cohesion in work teams because it decreases the level of conflict, anxiety among members and participation (Yoo and Alavi, 2001; Salas et al., 2015; Black et al., 2019) and increase satisfaction with the team (Tekleab et al., 2009). Most studies show that cohesion also favors productivity (Carless and De Paola, 2000; Carron and Brawley, 2000; Beal et al., 2003; Yang and Tang, 2004; Salas et al., 2015). In this sense, we can begin by indicating that Zaccaro et al. (2001), advocate that highly cohesive teams tend to show better performance in situations of adversity than those with low cohesion. This is because teams with high cohesion tend to be more united and develop better communication, are more committed to strive to achieve the established objectives and are therefore more decisive in the face of obstacles. On the contrary, when there is low cohesion, the members feel little motivation to participate and achieve the goals.

The positive relationship between cohesion and performance has also been defended in subsequent research; for example, Abrantes et al. (2020), in which they argue that cohesion serves as a mechanism to improve the performance of tenured teams. They point out that members develop cohesion when they feel connected and committed to each other to achieve objectives. In other words, each member fully identifies with the group and feels good being part of it; this generates trust, commitment and willingness to collaborate with their peers (Milliken and Martins, 1996; Taggar, 2002; Stahl et al., 2010). Into the context of multicultural teams, we understand that they need of knowledge sharing, personal bonds, social interactions with people from different cultures, and as a consequence, cohesion is even more relevant to achieve a successful team performance. Therefore, the following hypothesis is proposed:

*H3:* Cohesion is positively related to multicultural team performance.

## 5 Cultural diversity and multicultural team performance

Cultural diversity can be defined as the set of ways of thinking, attitudes, and values that characterize the team and the product of the mixture of national cultures of its members (Hofstede, 1980; Kim, 2017; Guzmán-Rodríguez et al., 2021; Stahl and Maznevski, 2021).

A frequently used model in cultural studies is the model of cultural dimensions of Hofstede (1980). This study defines “culture” as the system of values and beliefs of a society and establishes four cultural dimensions: Individualism vs. collectivism (i.e., degree to which society rewards individual vs. collective action); power distance (i.e., The extent to which people expect and agree that power should be shared unequally.); masculinity vs. femininity (i.e., societal preference for achievement, heroism, assertiveness, and material rewards for success); uncertainty avoidance (i.e., the degree to which society rewards individual vs. collective action.). Later on, three more dimensions are added to model of Hofstede (2001): long-term vs. short-term Orientation (i.e., a societies’ connection of the past with the current and future actions/challenges) and indulgence vs. restraint (i.e., Degree of freedom societal norms afford to citizens in fulfilling their human desires; Hofstede, 2001).

Although a high amount of literature on cultural diversity in teams has been performed (Matveev, 2017; Tabassi et al., 2019; Jones et al., 2020; Varma et al., 2020), it has produced mixed results (Tshetshema and Chan, 2020), because it not only contributes to providing new ideas and perspectives but can also produce adverse effects on group’s processes and performance (Dayan et al., 2017). The double-edged sword, the” nature of cultural diversity in teams, is supported by Stahl and Maznevski (2021). In this regard, they point out that cultural diversity would positively or negatively impact team outcome, depending on whether a team is composed of members from different countries or members from a single country.

Many studies focused on the benefits that cultural diversity brings to organizations. For instance, it has been widely studied that culturally heterogeneous teams can achieve higher levels of innovativeness and performance than culturally homogeneous teams (Feitosa et al., 2018; Stahl and Maznevski, 2021). Teams with members from various cultures can provide a broader range of perspectives, task-related knowledge, abilities, and skills (Gabelica and Popov, 2020).

Employees with diverse backgrounds having specific cultural knowledge and deployment of team member’s cross-cultural competence, diversity provides a creative advantage for teams to enhances successful outcomes (Jones et al., 2020) solve problems in different ways, and they may also have a higher tolerance for taking risks (Bertelsmann, 2018).

Previous literature based on Hofstede’s cultural model confirms the positive relationship between cultural diversity and creativity and innovation in economic terms (Williams and McGuire, 2010). When individuals have a clear collectivist orientation, there is low power distance and low uncertainty avoidance, all of which influence innovation. In other words, when human resources practices promote collectivism, it becomes easier for employees to feel identified with the organization, employees feel motivated to work as a team, because individual and collective interests coincide, and as a consequence, performance improves (Dahms and Kingkaew, 2019). On the contrary, when there is a clear individualistic orientation, this hinders trust among team members and as a consequence, causes a negative effect on team performance (Bouncken et al., 2016). Similarly, Vrânceanu and Iorgulescu (2016) conducted a comparative study according to Hofstede’s dimensions applied to Romanian service companies.

The results are opposite to expected in terms of the masculinity vs. femininity dimension, and similar in terms of power distance and tolerance for uncertainty.

Beyene et al. (2016) analyze firms in the textile manufacturing industry and find that high levels in all dimensions of cultural diversity negatively affect activities involved in innovative product development.

On the other hand, researchers conducted studies about cultural diversity and its adverse effects on team performance. In this regard, pessimistic perspectives are based on similarity-attraction theory (Byrne, 1997), suggesting that people prefer to interact with similar rather than dissimilar people. At the same time, social-identity theory (Tajfel and Turner, 2004) suggests that individuals prefer to classify themselves and others into certain social identity groups and that this identification has implications for advancing the interests of group members (Tajfel and Turner, 2004). In this case, team members would prefer peers from their own culture, which can make uneffective communication within teams, and as a consequence, be detrimental to team performance (Lisak et al., 2016). According to these theories, individual behaviors bring detrimental performance whene people display reluctant and uncomfortable reactions when interacting with colleagues who have different values and opposing personalities (Kim et al., 2017).

Therefore, the following hypotheses are proposed:

Cultural orientation is related to multicultural team performance, in such a way that:

*H4.1:* Collectivism is positively related to multicultural team performance.

*H4.2:* Power distance is negatively related to multicultural team performance.

*H4.3:* Uncertainty avoidance is positively related to multicultural team performance.

*H4.4:* Masculinity is negatively related to multicultural team performance.

## 6 Exploring differences between leader and team members and team performance

Differences in the views of stakeholders have implications for team performance (Kline, 2001). For instance, when work teams and their managers differ in their perceptions of variables such as group communication, organizational support (Tafvelin et al., 2017), or diversity climates (Mckay et al., 2009), there results a negative impact on team productivity. However, researchers have often treated these discrepancies in perceptions as “errors” (Bliese, 2000; Toegel and Conger, 2003) and have not studied them.

Gibson et al. (2009) develop in their research the concept of leader-team perceptual distance, considering the differences between a leader and a team in perceptions of the same social stimulus. They argued that the leader-team perceptual differences were related with a decrease in team performance. This relationship was explained on the basis of collective cognition of the team. Every team possesses cognitive properties (collective cognition) that are different from the sum of the cognitions of individual team members (Gobis et al., 2015). The lowered team performance due to leader-team perceptual differences is because these differences prevent the team from maximizing collective cognition and thus from reaching its full potential (Gibson et al., 2009).

Studies have used various terms such as “perceptual congruence” (Benlian, 2014), “perceptual fit” (Ostroff et al., 2005), and “perceptual similarity” (Gibson et al., 2009) in place of perceptual distance.

Few studies have analyzed the relation between different managerial and employee perspectives and organizational performance. When comparing leader-team perceptions with respect to communication, work performance, goal accomplishment, and organizational support, studies have found disagreement between leaders and their teams (Hatfield and Huseman, 1982; White et al., 1985; Engle and Lord, 1997; Heald et al., 1998; Hsiung and Tsai, 2009; Li and Thatcher, 2015). Further, when the disagreement between the leader and employees was high, it resulted in lower work performance (Ostroff et al., 2005; Fleenor et al., 2010). Conversely, lower perceptual differences were associated with increases in team performance (Gibson et al., 2009; Bashshur et al., 2011), and this effect is most substantial when a team’s perceptions are more favorable than the leader’s. Arguably then, team performance decreased when leader and team members disagreed (Bashshur et al., 2011).

It is the case of the study presented by McKay et al. (2009). They found that when both agreed positively in the perception of organizational diversity climate, in this case, related to the degree to which a firm is thought to utilize fair employee policies, the most outstanding performance happened, and when both agreed negatively in the perception about the issue, the lowest performance was shown in the company. It is also the case of Tafvelin et al. (2019), that indicated in their study that whether the leaders and their teams agree or not on perceptions of leadership has been found to impact follower well-being and performance. In their study they also proved that when leaders and followers agreed, leaders’safety leadership behaviors and followers’self-efficacy to give safety-related feedback improved. Cole et al. (2013) investigated the joint effect of leaders’ power distance values with their team’s power distance values on team performance. They found that incongruence between leaders’ and teams’ power distance values in either direction has an effect on team effectiveness mediated by justice perceptions.

Studies have also attempted to understand the effect of perceptual distance on team performance when a leader rated higher or lower than the team (Tafvelin et al., 2017). While Gibson et al. (2009) found that better team performance was achieved when the leader’s perceptions were slightly higher than that of the team, other studies found contrariwise. For instance, Cole et al. (2013) found that leader-team perceptual distance resulted in strong negative effect on the performance of the team when leader’s perception of power distance was higher than the perception of the team members. Similarly, Bashshur et al. (2011) suggested that team performance indicators were lowest when leaders perceived a higher support climate relative to the team. They explained the findings on grounds of passive leadership resulting due to higher leader perception due to which the leader fails to understand the needs of the team.

Several theoretical explanations have been extended to explain the outcomes on team performance resulting from leader-team perceptual differences or perceptual similarity. These include cognitive dissonance theory (Festinger, 1957), similarity-attraction paradigm (Byrne, 1997), consistency-based situational-strength (Meyer and Maltin, 2010), and organizational exchange theory (Blau, 1964, 1968).

A lack of perceptual agreement between the leader and the team is expected to weaken the obligation on the part of team members to reciprocate and perform at higher levels (Rhoades and Eisenberger, 2002; Chen et al., 2005). One explanation of perceptual differences between leader and the team is anchored in the relative difference in the position and power between the manager and the team (Patterson et al., 2004) due to which they have differential access to relevant information, different interpretations of that information and also different referents of comparison (Gibson et al., 2009).

Stahl and Maznevski (2021) reported that though sufficient literature was available on diversity in work teams (for, e.g., Bowers et al., 2000; Horwitz and Horwitz, 2007) as well as on the link between cultural diversity and team outcomes, the findings were at best equivocal. To date, no meta-analyses was found that specifically focused on cultural diversity and its effect on team performance *per se* (Stahl and Maznevski, 2021). Meta-analyses of research on work-group member diversity and team performance has not evidenced a relationship between the two. Hence, relatively little is yet understood regarding the mechanisms and factors that determine the effects of cultural diversity in teams. Researchers postulate that the effects of cultural diversity in teams are likely to be understood only when attention is directed toward contextual moderators and mediating mechanisms in research on multiculturally diverse teams (Mannix and Neale, 2005; Joshi and Roh, 2009). Thus, it was proposed by Stahl and Maznevski (2021) that cultural diversity does not have a direct impact on team performance. Rather, cultural diversity and its dimensions are likely to have an indirect effect on team performance, mediated by various process variables. Hence, it was important to bring together leadership and team dynamics to explain multicultural team performance. Stahl and Maznevski (2021) in their research retrospective sought to explore and identify the moderating factors in the relationship between cultural diversity and team performance. Since few studies have focused on moderating variables in studies of cultural differences and team performance (Stahl and Maznevski, 2021) in our study we focus on differences into the role played by the individuals (leader or team members) to explore a potential role as moderator in the relationship of individual characteristics, *viz.*, cultural sensitivity, adaptability, cohesion, and cultural sensitivity with multicultural team performance. Since our intention is to explore different views from leader and team members, we do not propouse a formal hypothesis about it. We suppouse a moderator role for role played into the teams, since leaders can reinforce some specific variables while team members reinforce another ones.

## 7 Methods

### 7.1 Sample

The sample used to test our hypotheses is composed of middle and senior managers working in internationalized Mexican and North American companies with subsidiaries in Mexico, South America, the United States, and the European Union.

Specifically, two criteria were used to select the companies in the sample, on the one hand, companies with international business activities related to exports, imports, mergers, and acquisitions; and on the other hand, companies with at least one subsidiary abroad. The suitability of these criteria is reflected in the work of Sullivan (1994), which indicates that these parameters can be good indicators of a company’s degree of internationalization.

Our sample consisted of a total of 180 companies. Human resources managers were contacted by email or telephone in order to know their availability to participate in our research. Additionally, we had the support of the Mexican Chamber of Commerce Industry in Monterrey, which contacted various organizations (and their human resource managers) linked to internationalized companies to obtain access to their databases for the application of the questionnaire, either electronically or in person.

### 7.2 Instrument

We collected data from team leaders and team members. To collect data, we used a survey questionnaire. Specifically, an online survey questionnaire. After 2 months collecting data, five hundred seventy questionnaires (570) were collected, but we discard 155 of them, because they did not fit the target profile or were not completed. Finally, we obtained a final sample of 415 participants, coming the 93% via online and 7% were completed fisically.

Demographic characteristics can be summarized it as follows: 111 (27%) responses were from team leaders, and 304 (73%) were team members. The average age was between 26 and 55 years old. In terms of gender, the sample was quite balanced, specifically we obtained response from 211 (51%) of male participants and 204 (49%) from females. Regarding nationalities, the predominant nationality was Mexican (291), representing the 70%, followed by 71 from Europe, representing the 17% of the sample, and the rest were from North American, Asian and Latin American (except Mexican) nationalities.

### 7.3 Measures

As we have mentioned above, the instrument for collecting information for all of the variables was a questionnaire, and responses were based on a five-point Likert scale, with responses ranging from “strongly disagree” to “strongly agree.” With the purpose to test the reliability and validity of our scales, we follow previous literature (Hair et al., 1999) and we performed a confirmatory factor analysis. Specifically, we performed it separately for each construct using SEM software, EQS 6.1. Results confirmed the reliability of our scales. Factor loading was at least 0.7 or close to, and the average extracted variance (AVE) higher than 0.5. Cronbach’s alpha score were also appropriate. Indicators of the goodness of fit are within the accepted limits (Muller, 1997).

Aditionally, following criteria of Fornell and Larcker (1981) to test discriminant validity, we found that all AVEs exceed square correlations (Table 1 summarizes AVEs results), supporting the discriminant validity among our variables. Information about items used, factor loadings, R^2^, the goodnesses of confirmatory factor analysis (CFA) to each variable is shown in Appendix I.

**Table 1:** Composite reliability (CR), average variance extracted (AVE), and square correlations between variables.

CR	0.756	0.799	0.920	0.342	0.86	0.870	0.80	0.90
AVE	0.789	0.718	0.692	0.802	0.801	0.779	0.776	0.801
	1.	2.	3.	4.	5.	6.	7.	8.
1. Cultural Sensibility	—							
2. Adaptability (Team adjustment)	0.345	—						
3. Cohesiveness	0.015	0.066	—					
4. Collectivism	0.021	0.028	0.030	—				
5. Masculinity	0.023	0.007	0.010	0.008	—			
6. Power Distance	0.034	0.002	0.017	0.006	0.467	—		
7. Uncertainty	0.034	0.019	0.01	0.012	0.006	0.001	—	
8. Multicultural team performance	0.093	0.133	0.177	0.035	0.002	0.003	0.043	—

#### 7.3.1 Multicultural team performance

According to Kinnebrew (2011), measurement of team performance is limited due to they carry it out a certain degree of objectivity, and following her recommendation, we used the measurement scale called “team productivity index” by Kirkman and Rosen (1999) and modified by Kinnebrew (2011) because, it is considered a reliable measure that can be applied to cultural contexts. It consists of six items (see Appendix I).

#### 7.3.2 Cultural sensibility

Cultural sensitivity is the ability to empathize with people’s interests, thoughts, values, and ideas from different cultures. To measure cultural sensibility variable, we used the items from the managerial, cultural flexibility scale obtained from previous studies by Puck et al. (2008) and consists of four items (see Appendix I).

#### 7.3.3 Adaptability

Adaptability to the multicultural team measures the degree of comfort a team member or team leader has when working with the rest of the members from cultures other than their own (Black et al., 1991; Bhaskar-Shrinivas et al., 2005). The three items (see Appendix I) to measure this variable comes from the scale of “adaptation to a multicultural context” by Puck et al. (2008).

#### 7.3.4 Cohesion

Team cohesion is the link between the multicultural team members, their commitment and integration with the team’s goals (Kozlowski and Ilgen, 2006). To measure this variable, cohesiveness scale of Chidambaram (1996) is used. It is measured through four items (see Appendix I).

#### 7.3.5 Cultural diversity

To measure cultural diversity, we used four dimensions or theoretical constructs distinguished by Hofstede (1980, 2001) and House et al. (2004). They are the collectivist orientation (as opposed to the individualist), the distance of power, the orientation toward masculinity (gender roles) and the avoidance of uncertainty (aversion as opposed to tolerance). For each dimension, a set of items was proposed to the respondents. Specifically, six items have been used for collectivist orientation, for power distance, five in orientation toward masculinity and five for uncertainty avoidance. Items can be checked into the Appendix I.

#### 7.3.6 Leader or team member role

We designed “1” if the position was the team leader, and “2” if the position was the team member.

#### 7.3.7 Control variables

Gender: Gender of participants were identified as 1 = male, 2 = female. Age: Age of participants were ranked using the following categories: (1) from 18 to 25, (2) between 26 and 35, (3) between 36 and 45 years, (4) between 45 and 55 years, and (5) more than 55 years. International experience: We have defined previous international experience as the period of time measured in years that the member has participated in an international work experience, because, following Schneider and De Meyer (1991), the person who has said experience contributes valuable knowledge of global management in addition to a variety of different opinions due to their interaction with international environments (Schneider and De Meyer, 1991). In this regard, participants responded regarding working international experience and answered one of the following options (1) none, (2) less than 2 years, (3) between 2 and 3 years, (4) between 3 and 5 years, and (5) more than 5 years. Previous experience in multicultural teams: Regarding previous experience in multicultural teams, participants responded to the sentence regarding experience working multicultural teams and by choosing one of the following options: (1) none, (2) less than 2 years, (3) between 2 and 3 years, (4) between 3 and 5, and (5) more than 5 years.

## 8 Results

Table 2 shows the leading statistics and correlations. The majority of the relationships at the correlational level are as expected. Multicultural team performance was significant and positively correlated to cultural sensibility, multicultural team adjustment, cohesiveness, collectivism, and uncertainty avoidance. Correlations between multicultural team performance and power distance and masculinity were negative but not significant.

**Table 2:** Main statistics and correlations.^a^

	Mean	SD	1.	2.	3.	4.	5.	6.	7.	8.	9.	10.	11.	12.	13.
1. Gender	1.49	0.500	1												
2. Age	2.95	1.07	−0.127**	1											
3. International experience	3.11	1.32	−0.173**	0.386**	1										
4. Previous experience	3.05	1.23	−0.116*	0.337**	0.437**	1									
5. Team role	1.73	0.443	0.232**	−0.179**	−0.196**	−0.141**	1								
6. Cultural sensibility	4.61	0.603	−0.057	−0.003	0.114*	0.077	−0.027	**(0.90)**							
7. Adaptability (Team adjustment)	4.50	0.595	−0.015	0.070	0.163**	0.190**	−0.036	0.588**	**(0.86)**						
8. Cohesiveness	3.79	0.820	−0.056	−0.031	−0.030	0.039	−0.049	0.126*	0.257**	**(0.87)**					
9. Collectivism	3.80	0.855	−0.143**	−0.013	0.014	0.030	−0.047	0.146**	0.168**	0.176**	**(0.82)**				
10. Power distance	2.35	0.892	−0.103*	−0.114*	−0.063	−0.102*	0.071	−0.187**	−0.045	0.131**	0.078	**(0.86)**			
11. Uncertainty avoidance	4.15	0.662	0.002	−0.061	−0.123*	−0.061	−0.075	0.186**	0.139**	0.100*	0.112*	0.040	**(0.85)**		
12. Masculinity	1.92	1.01	−0.138**	−0.148**	−0.054	−0.139**	0.037	−0.152**	−0.084	0.104*	0.094	0.684**	0.078	**(0.91)**	
13. Multicultural team performance	4.09	0.543	−0.074	−0.004	0.001	0.111*	−0.112*	0.306**	0.365**	0.421**	0.189**	−0.059	0.208**	−0.047	**(0.90)**

*n* = 415. ^*^p < 0.05; ^**^p < 0.01. When appropriate Cronbach’s alpha coefficients are reported in parentheses on the diagonal.

To test our hypotheses, hierarchical regressions were performed. Specifically, we introduced control variables in a first step (model 1), main variables in a second step (model 2), and finally, interaction terms after centring variables were introduced (model 3). The results are shown in Table 3. Hypothesis 1 establishes the positive relationship between cultural sensitivity and multicultural team performance, and our results support the theoretical prediction (*β* = 0.100*). Same regression results for H2 and H3. Specifically, to the relationship between adaptability and multicultural team performance, we obtained a statistically significant coefficient (*β* = 0.160); to the relationship between cohesiveness and multicultural team performance, we also obtained a statistically significant coefficient (*β* = 0.225), finding support to our hypotheses H2 & H3.

**Table 3:** Hierarchical regression results for hypotheses.

	Coefficients model		
	*Model β*	*Model 2 β*	*Model 3 β*
*Control variables*			
Gender	−0.079	−0.039	−0.033
Age	−0.019	−0.013	−0.018
International experience	−0.024	−0.026	−0.029
Previous experience	0.062**	0.033	**0.039+**
*Main variables*			
Cultural sensibility		0.100*	0.533*
Adaptability (Team adjustment)		0.160**	0.531*
Cohesiveness		0.225**	−0.157
Collectivism		0.043	0.135
Power distance		−0.044	−0.056
Uncertainty avoidance		0.095	−0.018
Masculinity		−0.009	−0.101
Team role		−0.062	1.16*
*Interaction terms*			
Team role × Cultural sensibility			−0.244
Team role × Adaptability			−0.240*
Team role × Cohesiveness			0.210**
Team role × Collectivism			−0.053
Team role × Power distance			0.014
Team role × Uncertainty avoidance			0.058
Team role × Masculinity			0.049
*R* ^2^	0.012	0.28	0.329
Δ*F*	2.2	20,13**	5.17**

^*^p < 0.05; ^**^p < 0.01. Dependent variable: multicultural team performance.

Our theoretical assumptions predicted different positive and negative relationships between cultural orientation and multicultural team performance (H4). Unfortunately, our data showed no statistical significance to any of the relationships between dimensions of cultural orientation and multicultural team performance, at least, not direct relationships.

**Figure 1:**
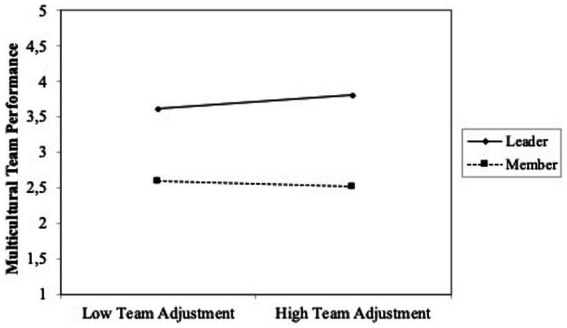
Plot interaction team role (Leader/ member) and adapability (Team adjustment) on multicutural team performance.

**Figure 2:**
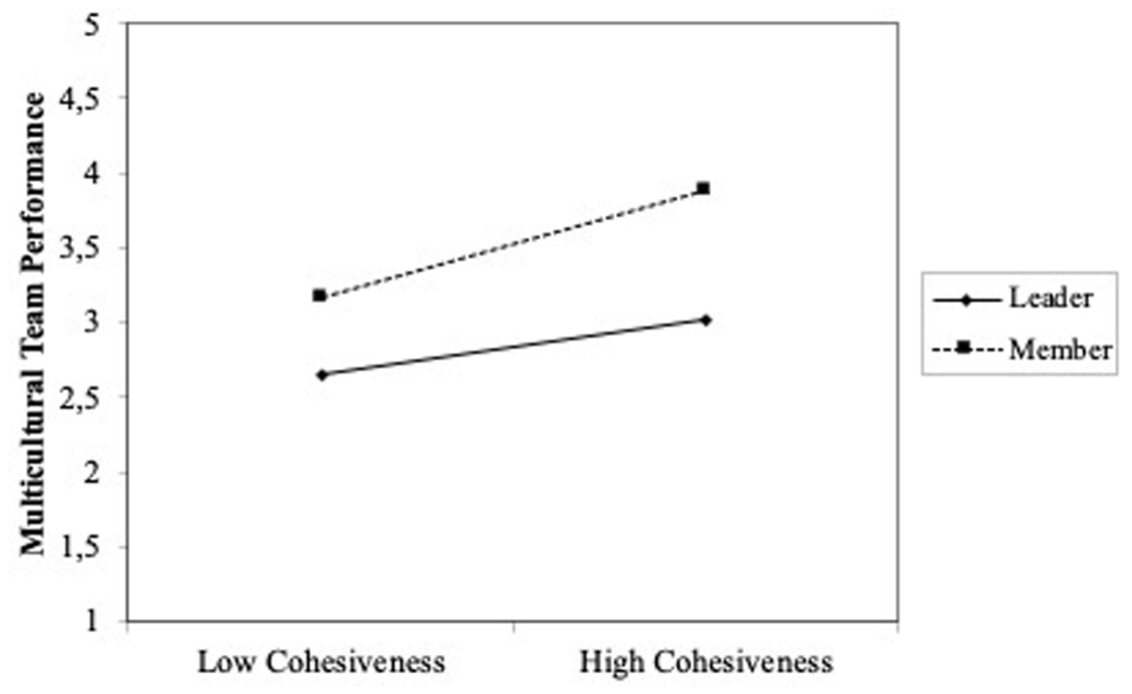
Plot interaction team role (Leader/ member) and cohesiveness (Team adjustment) on multicutural team performance.

Finally, with the aim to explore if the role played by individuals could introduce differences into our results, we introduced interactions terms into the regression (model 3, Table 3). We found statistical significance for two of the relations tested. Specifically, we found that the role played by individuals into the team (leader or team member) interacts with main variables so that adaptability to the team and cohesiveness change depending on if the role played is team leader or team member. To interpret the meaning of the statistically significant interactions, we plotted team role about adaptability and team role about “Cohesiveness” (Figures 1, 2; Aiken et al., 1991). High and low levels of adaptability (team adjustment) and cohesiveness were defined as one standard deviation above and below the mean, respectively. In Figure 1 can be observed that when individuals play a team leader role, adaptability (team adjustment) is significatively related to multicultural team performance. Figure 2 shows that cohesiveness is significatively related to multicultural team performance when individuals play team member role.

## 9 Discussion and conclusion

The main purpose of this empirical study was to analyze the impact of individual characteristics as cultural sensitivity, adaptability, cohesion, and cultural diversity on the multicultural team performance. The findings indicate the existence of a positive relationship between cultural sensitivity, adaptability, cohesion, and team performance. Cultural diversity did not show a significant impact on team performance.

It seems that the ability to understand people’s point of view from different cultures, to accept, understand and appreciate cultural differences enhances intercultural communication quality (Peng, 2006; Liu-Farrer, 2014) minimizes cultural differences within team members (Segura-Robles and Parra-González, 2019) and improves performance (Deardorff, 2006; Ramthun and Matkin, 2012). The results show congruence with previous research (Puck et al., 2008; Chua et al., 2012; Segura-Robles and Parra-González, 2019; Moradi, 2020) who pointed out that cultural sensitivity positively influences the performance of multicultural teams. This means that team members and leaders consider that in order to achieve multicultural team performance, it is not only important to be aware of the differences in perspectives and approaches caused by cultural diversity, but also, it is crucial to have the ability to empathize with those values and ideas. This ability to empathize encloses a deep understanding of an individual’s feelings from different cultures, their points of view, interests, values, and beliefs.

The foregoing leads to suggesting the importance of designing strategies aimed at, on the one hand, promoting cultural sensitivity that imply an open attitude toward constructive discussion, the absence of prejudices and tolerance regarding other ways of thinking, and on the other hand, to create awareness in the members about the importance of effective communication in the team. All of this implies expressing their opinions clearly and directly with the rest of the team. We also suggest that developing trust, honesty, tolerance, effective communication, and a genuine interest for collaboration are strategies that may help to foster cultural sensitivity. Additionally, developing trust, honesty, tolerance fosters an effective communication between team leaders and members along with a genuine interest for collaboration may be a good strategy to enhance cultural sensitivity.

Prior literature suggests that adaptability is considered one of the strategic global talent development skills required to do business effectively (Bayraktar, 2019) based on people to adapt successfully to change deploys excellence in problem-solving, uncertainty management, stress, crisis, new learning related to people, culture, and environment (Pulakos et al., 2000; Park and Park, 2019). For instance, our findings showed that, team members consider this adaptability as important due to its impact on team performance, this result finds consistency with previous studies that establish a positive relationship between both variables (Pulakos et al., 2000; Puck et al., 2008; Beus et al., 2014; Park and Park, 2019; Peltokorpi and Zhang, 2020). One possible reason to explain this is that new members go through a kind of adaptation or learning curve when joining the team and, because their peers come from different cultures, if you do not have the ability to adapt, understand a different way of doing things, and be willing to work on it, the culture clash within different values and mental schemes can be detrimental to the group performance.

Regarding team cohesion, our results support previously conducted research that shows that in cohesive teams, members feel connected and committed to each other and collaborate to achieve objectives. In the same way, each member fully identifies with the group and feels good being part of it; this generates trust, commitment, and willingness to collaborate with their peers (Milliken and Martins, 1996; Stahl et al., 2010) to improves performance (Zaccaro et al., 2001; Rico et al., 2012; Salas et al., 2015; Abrantes et al., 2020). This indicates that for team members it is very important to establish links within colleges in the team because this enhances the sense of belonging, team identification feeling and fosters motivation to contribute to a common goal. Taking the above arguments into account, we recommend establishing training programs to encourage leaders to become aware of the importance that cohesion is for team members.

Literature maintains that cultural diversity in teams is a source of benefits based on the mix of knowledge, experience, values and perspectives that enhances the team and therefore, it is considered as a path to improve team performance. In this regard, and contrary to what we expected, the results of both groups did not show significant evidence of this relationship in any of the four Hofstede’s dimensions. In other words, both leaders and members agree that an orientation to collaboration and submission of individual interests for the team goals (e.g., collectivism), expectation regarding importance of positions within team hierarchy (e.g., power distance dimension), preference for achievement, competition, confrontation and individual goals and material rewards for success (e.g., masculinity dimension), and the preference to avoid working under unplanned situations or in the absence of formal rules and structures (e.g., Uncertainty avoidance) are factors that do not significantly influence the team performance. Our findings exploring the effects of cultural diversity of multicultural team performace are slight similar than previous work on multicultural team innovation (Guzmán-Rodríguez et al., 2021) although in that case, Guzman-Rodriguez and colleages found than only the power distance orientation affected positively to the team innovation.

One possible argument to explain the above is because the predominant culture is Mexican one, and considering that it is a culture oriented to teamwork and to establish links with each other, the members feel motivated and committed to contribute significantly to the performance, therefore, they hardly express opinions contrary to the majority in order not to cause conflicts and maintain group harmony. This argument finds consistency with studies that consider due to the nature of cultural diversity may has a “double-edged sword” so, it would positively or negatively impact team outcome, depending on level of diversity within the teams.

A second argument to explain this lack of relationship between cultural diversity and the performance of teams for leaders and members may be since the companies chosen for the study have a long way to go in terms of the management of cultural diversity in teams, teams have support mechanisms that help their proper functioning and successful management of diversity.

Finally, based on the exploration and analysis of the moderating role of the role played by individuals (leaders or team members) into the team, significant differences have been found between the perception of the members and the perception of the leaders about the concepts that affect team performance. For instance, according to team members’ perception, cohesion is a variable that positively influences performance. Conversely, leaders differ in the above because they do not perceive cohesion as a variable that determines performance. The foregoing suggests that, perhaps, taking into consideration that the Mexican culture is the predominant one into the sample, and, that it is characterized by having a high distance of powers, the differences in status between the leader and the members are clearly defined, therefore, it can be expected that feeling identified with the team and with a common goal is more important for the members since, based, in some way, they are only those who work more closely, considering that the leader at a higher level due to the marked difference in status.

On the other hand, leaders consider that the ability to adapt to the team (e.g., adaptability) is a factor that determines the achievement of performance, this could be because when leaders are assigned to a team or vice versa, perhaps adaptation is one of the main steps as part of the organizational socialization, therefore, it entails a key in successful collaboration. This shows the need, in the first place, to identify the needs, expectations, and motivations of each team leader and members separately, to establish mechanisms for minimizing those differences in such a way that both individual and team’s goals can be achieved successfully. This result supports previous investigations that point out that leader-team perceptual differences are detrimental to team performance (Gibson et al., 2009).

This study contributes to the literature because it provides a deeper understanding of the internal mechanism of multicultural teams and their performance work. This will give companies the tools to face a growing need for internationalization more competitively.

The results indicate that managers must consider cultural sensitivity, adaptability, and cohesion as individual characteristics of the members in the recruitment and training programs in order to achieve a multicultural team’s successful performance.

Training programs must include a formal development of adaptation skills to work with people from different cultures, and to strengthen cultural sensitivity in team members and team leaders to prepare them to manage differences originated by the diversity of perspectives, values, and experiences based on team members’ culture.

Finally, this study makes it possible to identify a clear difference between the perceptions of leaders and the members about the factors that influence team performance. This is a call to pay more attention to the needs of both groups and to everything in the design and implementation of mechanisms to eliminate those differences in perceptions or to minimize them as much as it could be possible.

## Data availability statement

The raw data supporting the conclusions of this article will be made available by the authors, without undue reservation.

## Ethics statement

Ethical review and approval was not required for the study on human participants in accordance with the local legislation and institutional requirements. Written informed consent from the participants was not required to participate in this study in accordance with the national legislation and the institutional requirements.

## Author contributions

LG-R: Investigation, Writing – original draft, Writing – review & editing. AA-E: Investigation, Writing – original draft, Writing – review & editing. TA: Investigation, Writing – original draft, Writing – review & editing. MB-B: Investigation, Writing – original draft, Writing – review & editing.

## Appendix

Appendix I

Items and Results for CFA for each of the measures.

**Table:**

Variables	Items	Factor loadings	R^2^	CFA indices
Cultural sensibility	1. I feel comfortable getting in contact with new cultures 2. I try to get a better understanding of people from other cultures. 3. I try to build good relationships with colleagues from other cultures. 4. I try to understand the different norms, values and beliefs of different cultures.	0.787	0.620	**Satorra-Bentler χ**^**2**^ = 48.77 (*p* = 0.03; Degree of freedom = 32) **GFI** = 0.97 AGFI = 0.95 **CFI** = 0.97 **RMSEA** = 0.036 (0.012, 0.055)
		0.832	0.691	
		0.878	0.770	
		0.862	0.743	
Adaptability (Team Adjustment)	1. Working with co-workers from different cultures 2. The intercultural context in your team 3. Working with different cultures at once.	0.874	0.765	
		0.759	0.576	
		0.836	0.699	
Cohesiveness	1. I feel that I am a part of the team 2. My team works together better than most teams on which I have worked 3. My teammates and I help each other better than most other teams on which I have worked 4. My teammates and I get along better than most other teams on which I have worked.	Removed	0.628	
		0.792	0.803	
		0.896	0.807	
		0.807		
Team Performance	1. My team meets or exceeds its goals 2. My team completes its tasks on time 3. My team makes sure that products and services meet or exceed quality standards 4. My team responds quickly when problems come up 5. My team is a productive team 6. My team successfully solves problems that slow down our work	0.706	0.503	**Satorra-Bentler χ**^**2**^ = 37.25(*p* = 0.000; Df = 9) (**GFI**) = 0.94 AGFI = 0.87 (**CFI**) = 0.92 (**RMSEA**) = 0.087 (0.059, 0.117)
		0.711	0.506	
		0.798	0.636	
		0.811	0.657	
		0.857	0.735	
		0.779	0.606	
Collectivism	1. Group welfare is more important than individual rewards 2. Group success is more important than individual success 3. Being accepted by members of your work group is very important 4. Employees should only pursue their goals after considering the welfare of the group 5. Managers should encourage group loyalty even if individual goals suffer 6. Individuals may be expected to give up their goals in order to benefit group success	0.878	0.770	**Satorra-Bentler χ**^**2**^ = 205.42 (p = 0.000; Df = 129) **GFI** = 0.93 **AGFI** = 0.91 **CFI** = 0.97 **RMSEA** = 0.038 (0.028, 0.047)
		0.789.		
		Removed		
		Removed	0.622	
		Removed		
		Removed		
Masculinity	1. Meetings are usually run more effectively when they are chaired by a man 2. It is more important for men to have a professional career than it is for women to have a professional career. 3. Men usually solve problems with logical analysis; women usually solve problems with intuition 4. Solving organizational problems usually requires an active forcible approach, which is typical of men 5. It is preferable to have a man in high level position rather than a woman	0.708	0.501	
		0.853	0.728	
		0.772	0.595	
		0.920	0.846	
		0.896	0.803	
Power Distance	1. Managers should make most decisions without consulting subordinates 2. It is frequently necessary for a manager to use authority and power when dealing with subordinates 3. Managers should seldom ask for the opinions of employees 4. Managers should avoid of-the-job social contacts with employees 5. Employees should *not* disagree with management decisions 6. Managers should not delegate important tasks to employees	0.709	0.502	
		0.578	0.334	
		0.668	0.446	
		0.811	0.658	
		0.756	0.572	
		0.753	0.568	
Uncertainty Avoid	1. It is important to have job requirements and instructions spelled out in detail so that employees always know what they are expected to do 2. Managers expect employees to closely follow instructions and procedures 3. Rules and regulations are important because they inform employees what the organization expects of them 4. Standard operating procedures are helpful to employees on the job 5. Instructions for operations are important for employees on the job.	0.583	0.340	
		0.712	0.507	
		0.836	0.699	
		0.802	0.642	
		0.789	0.622	
